# Supplementary material for: Mechanisms governing the pioneering and redistribution capabilities of the non-classical pioneer PU.1
Source: Nat Commun. 2020 Jan 21;11:402. doi: 10.1038/s41467-019-13960-2 (PMC6972792; doi:10.1038/s41467-019-13960-2)
Supplement: Supplementary file 7 — Source data [file 41467_2019_13960_MOESM7_ESM.zip › Source_Data/FigureS3/FigureS3J_MotifScanOutput/homerResults/motif19.similar.html]

motif19

## Information for motif19

T
A
G
C
G
C
A
T
C
G
A
T
C
G
T
A
T
C
A
G
G
C
T
A
G
A
C
T
G
T
C
A
G
T
C
A
C
T
G
A
G
T
A
C
G
C
T
A
  
Reverse Opposite:  

C
G
A
T
C
A
T
G
G
A
C
T
A
C
G
T
C
A
G
T
C
T
G
A
C
G
A
T
A
G
T
C
G
C
A
T
G
C
T
A
C
G
T
A
A
T
C
G
  

|  |  |
| --- | --- |
| p-value: | 1e-61 |
| log p-value: | -1.411e+02 |
| Information Content per bp: | 1.629 |
| Number of Target Sequences with motif | 1317.0 |
| Percentage of Target Sequences with motif | 9.98% |
| Number of Background Sequences with motif | 2040.9 |
| Percentage of Background Sequences with motif | 5.59% |
| Average Position of motif in Targets | 378.3 +/- 349.9bp |
| Average Position of motif in Background | 323.8 +/- 198.8bp |
| Strand Bias (log2 ratio + to - strand density) | -0.1 |
| Multiplicity (# of sites on avg that occur together) | 1.06 |
| Motif File: | file (matrix) reverse opposite |

### Similar de novo motifs found

|  |  |  |  |  |  |  |  |
| --- | --- | --- | --- | --- | --- | --- | --- |
| Rank | Match Score | Redundant Motif | P-value | log P-value | % of Targets | % of Background | Motif file |
| 1 | 0.882 | C T G A T C A G G T C A A G C T G C T A C T G A T C G A T G A C | 1e-61 | -140.578159 | 28.85% | 21.61% | motif file (matrix) |
| 2 | 0.767 | G C T A A C T G C G T A A C G T C G T A C G T A T C A G | 1e-47 | -108.245297 | 31.10% | 24.55% | motif file (matrix) |
| 3 | 0.760 | T A G C A G C T C G A T C G T A A G C T A T G C G C A T G T A C C G T A | 1e-46 | -106.303581 | 11.74% | 7.52% | motif file (matrix) |
| 4 | 0.810 | A C G T A C T G A C G T A C G T A C G T C G T A A C G T A G C T A C G T A G C T | 1e-25 | -58.377982 | 3.58% | 1.89% | motif file (matrix) |
